# Modulation of sensorimotor cortical oscillations in athletes with yips

**DOI:** 10.1038/s41598-021-89947-1

**Published:** 2021-05-14

**Authors:** Tatsunori Watanabe, Kiyoshi Yoshioka, Kojiro Matsushita, Shin Ishihara

**Affiliations:** 1 Department of Sensorimotor Neuroscience, Graduate School of Biomedical and Health Sciences, Hiroshima University, Hiroshima, Japan; 2 Department of Physical Therapy, Nagoya University Graduate School of Medicine, Nagoya, Japan; 3 Institute for Research on Productive Aging, Kobe, Japan; 4 Department of Mechanical Engineering, Faculty of Engineering, Gifu University, Gifu, Japan; 5 Havana Trainers Room, Tokyo, Japan

**Keywords:** Dystonia, Movement disorders, Motor control, Neurophysiology

## Abstract

The yips, an involuntary movement impediment that affects performance in skilled athletes, is commonly described as a form of task-specific focal dystonia or as a disorder lying on a continuum with focal dystonia at one end (neurological) and chocking under pressure at the other (psychological). However, its etiology has been remained to be elucidated. In order to understand sensorimotor cortical activity associated with this movement disorder, we examined electroencephalographic oscillations over the bilateral sensorimotor areas during a precision force task in athletes with yips, and compared them with age-, sex-, and years of experience-matched controls. Alpha-band event-related desynchronization (ERD), that occurs during movement execution, was greater in athlete with yips as compared to controls when increasing force output to match a target but not when adjusting the force at around the target. Event-related synchronization that occurs after movement termination was also greater in athletes with yips. There was no significant difference in task performance between groups. The enhanced ERD is suggested to be attributed to dysfunction of inhibitory system or increased allocation of attention to the body part used during the task. Our findings indicate that sensorimotor cortical oscillatory response is increased during movement initiation in athletes with yips.

## Introduction

Loss of motor skills in professional athletes can have consequences for their career. Even in experienced amateur athletes, it could lead to deprivation of enjoyment of the skillful sport activity. The yips is one of those motor phenomena that have a large impact on performance of athletes and was first defined as an involuntary movement affecting golfers with low handicaps during putting or chipping^1^. With recent evidence showing the yips in other sports^2–4^, however, it was redefined as “a psycho-neuromuscular impediment affecting the execution of fine motor skills during sporting performance”^5,6^, characterized by twitches, jerks, freezing, and tremors of a planned movement. Although the yips has been reported to be observed in a relatively large number of golfers (17–48%)^1,7,8^ and has been apparent in athletes in other sports^2–4^, its scientific research has been scarce so far.

The etiology of the yips is thought to be multifactorial and has been remained unclear. The most well-known explanation is a continuum model proposed by Smith and colleagues^7,9^. In this model, the yips is placed on a continuum between task-specific focal dystonia (neurological) and choking under pressure (psychological). The task-specific focal dystonia is a movement disorder characterized by abnormal posturing and tremors during motor tasks^10^, and well-recognized task-specific focal dystonia includes writer’s cramp and musician’s dystonia^11^. Meanwhile, choking under pressure is defined as a perceived insufficiency in the individual resources to meet situational demands occurring due to increased anxiety^12^. Athletes suffering from the yips are considered to reside somewhere on the continuum^7,9^, although the exact location is hardly determined. On the other hand, some other research argues that the yips is a task-specific form of focal dystonia that is worsened by psychological elements^13^, or that psychological elements are triggering factors for the task-specific focal dystonia or the yips^14^. Contradictory results in studies examining psychological profiles in athletes with yips^5,13,15–17^ and the existence of symptoms similar to task-specific focal dystonia (e.g., co-contraction) may support this argument^15,18^.

The pathophysiological mechanisms of task-specific focal dystonia are not completely understood, but one of the most promising to explain it is dysfunction of inhibitory system as evidenced in several neurophysiological studies. For example, functional magnetic resonance imaging (fMRI) and positron emission tomography studies have reported that focal dystonia was associated with hyperactivity of motor-related cortical areas^19–21^. Furthermore, in transcranial magnetic stimulation (TMS) studies, motor cortical excitability was increased, and short intracortical inhibition was reduced in individuals with task-specific focal dystonia^22–26^. The reduced inhibition has been proposed to impair surrounded inhibition, a mechanism whereby activations of task-irrelevant muscles are actively inhibited during a motor task^27^. In contrast to these consistent brain imaging and TMS studies’ outcomes, findings regarding cortical oscillatory activity have been equivocal. For instance, using a self-paced movement task, Deuschl and colleagues showed that movement-related cortical potential (MRCP) preceding the movement was reduced over the central area in individuals with task-specific focal dystonia^28^. Also, Toro and colleagues reported that event-related desynchronization (ERD) of 20–30 Hz band oscillations over the central areas, which can reflect activation of the sensorimotor area^29^, was smaller just prior to and just after the time of self-paced movement onset in focal dystonia group^30^. Similarly, beta-band oscillatory power decrease was shown to be reduced during movements in individuals with task-specific focal dystonia^31^. On the other hand, Yazawa and colleagues demonstrated that MRCP preceding a self-paced voluntary contraction was greater over the ipsilateral central area in focal dystonia than control group^32^. Furthermore, Tseng and colleagues showed that sensorimotor beta-band event-related synchronization (ERS) following a self-paced finger movement termination, which can reflect inhibition of the motor cortex^33^, was smaller in focal dystonia than control group whereas beta-band ERD following the movement onset was not different^34^. Nevertheless, current evidence suggests abnormal sensorimotor cortical activity in individuals with task-specific focal dystonia. However, it is currently unknown whether cortical oscillatory activity in athletes with yips is different from those without yips.

Accordingly, in this study we compared the ERD/ERS during a fine force control task between athletes with and without yips. We hypothesized that: (1) alpha/beta-band ERD would be exaggerated when attempting to precisely control force in athletes with yips, (2) alpha/beta-band ERS following the termination of force control would be diminished in athletes with yips, and (3) modulations in these oscillatory activities would be greater when higher precision is required.

## Methods

### Subjects

In this cross-sectional study, ten athletes with yips (mean age = 25.8 ± 5.8 years, all male) and ten age-, sex-, and years of experience-matched controls (mean age = 24.4 ± 5.1 years) participated in the experiment. The yips was defined as “an involuntary movement impeding athletic performance that had been proficient and automated through practice,” and its presence was confirmed by two of the authors (one sport trainer and one physical therapist) who have more than 10 years of experience treating athletes with yips, as there is no conclusive diagnostic test^15^. Characteristics of the participants are shown in Table 1. None of them was under treatment at the time of experiment. Exclusion criterion was a history of psychiatric and neurological disorders other than the yips. All participants had normal or corrected-to-normal vision and were right-handed by self-report. Each subject provided written informed consent prior to the study. This study was approved by the ethics committee of Nagoya University and conducted in accordance with the Declaration of Helsinki, and followed STROBE (STrengthening the Reporting of OBservational studies in Epidemiology) guidelines. Also, this trial was registered with the University Hospital Medical Information Network Clinical Trials Registry as UMIN000042062 (date of registration: 09/10/2020).

**Table 1:** Characteristics of athletes with yips.

Number	Age	Sport	Years exp	Affected side	Affected movement	Years affected
1	24	Baseball	12	Right	Throw	5
2	20	Baseball	11	Right	Throw	7
3	35	Baseball	28	Right	Throw	16
4	33	Baseball	9	Right	Throw	5
5	30	Baseball	10	Right	Throw	2
6	30	Baseball	20	Right	Throw	11
7	25	Baseball	12	Right	Throw	10
8	21	Baseball	16	Right	Throw	6
9	19	Baseball	10	Right	Throw	2
10	21	Badminton	10	Right	Serve	3

### Experimental procedure

The subject sat on a chair in front of a table on which a PC monitor was set (eye to monitor distance of 0.6 m) and was asked to lateral-pinch grip a force transducer (Tech Gihan, Kyoto, Japan) using the thumb and index finger. The subjects with yips used the affected side, and the control subjects used the side that was used in playing his sport (right for all subjects).

Prior to an experimental task, we determined maximal voluntary isometric force (MVF). The subject gradually increased force over 3 s and kept maximum for 2–3 s. Verbal encouragement to make maximum effort was provided to the subject. The highest force over three contraction trials was adopted as the MVF.

**Figure 1:**
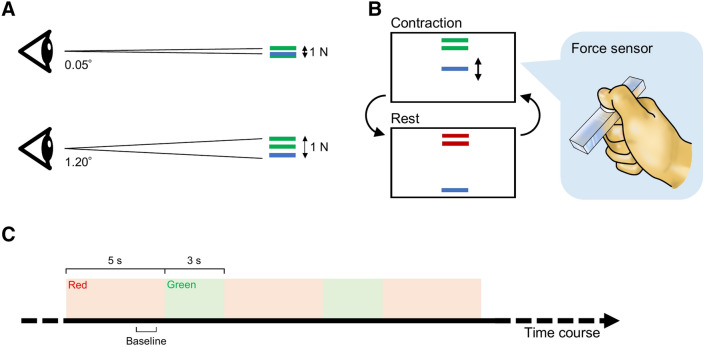
Visual gain manipulation and schema of experimental setup and paradigm. The spatial amplitude of visual feedback was larger at higher visual gain (**A**). The subject produced force to place the blue bar between two green bars as accurately and steadily as possible and stopped contraction when color of these bars turned red (**B**). The bars were red for 5 s and green for 3 s. Event-related de/synchronization was computed using a baseline interval of − 1500 to − 500 ms with respect to force onset (**C**).

The experimental task was similar to that in our previous study^35^. During the task, three horizontal parallel bars were displayed on the PC monitor (Fig. 1). One of them moved horizontally in real time according to the contraction force produced by the subject (force bar). The other two bars, that were separated by the length of the smaller side of the bar, stayed at the same position (target bars). Color of these target bars changed between green and red. The subject was asked to begin producing force as soon as the target bars turned green and to place the blue force bar between two target bars as accurately and steadily as possible. He was required to stop producing force as soon as the target bars turned red. The target bars were green for 3 s and red for 5 s, and the target force level was set at 15% MVF. A customized LabVIEW program (National Instruments, Austin, TX, USA) was used to display the bars and collect 20 Hz low-pass filtered force data.

We adjusted the level of precision required in the task by manipulating visual gain (Fig. 1). It was done by changing the height of the force fluctuation on the monitor, as the eye-to-monitor distance remained constant. One could see small deviations from the target with the higher visual gain, requiring the higher precision. We computed the visual angle using the following equation^35–40^:

$$ {\it visual}\;{\it angle} = 2\tan^{ - 1} \left( \frac{h}{d} \right), $$

where *h* is half of the force fluctuation, and *d* is the eye-to-monitor distance. In this study we used visual gains of 0.05° and 1.20°, because behavioral force parameters were shown to be mainly different between below and above 1°^41^.

Each subject performed three blocks of 20 trials at each visual gain, and a sufficient rest was provided between blocks.

### Electroencephalography measurements

Electroencephalography (EEG) was recorded at a sampling rate of 500 Hz using active electrodes (Polymate Mini AP108, Miyuki Giken, Tokyo, Japan). The electrodes were placed at C3 and C4 according to the international 10–20 system. The ground and reference electrodes were located at the forehead and left earlobe, respectively. During the experimental task, the subject was asked to keep their eyes on the target bars and also to avoid unnecessary eye movements to reduce artifacts. The electrical impedance was kept below 10 kΩ.

### Data analysis

Data analysis was similar to that in our previous study^35^ and was completed in Matlab (MathWorks, MA, USA). The force data were low-pass filtered at 15 Hz (4th-order Butterworth filter) and converted to the rate of force change (N/s). The force onset and offset were defined as the first time point at which the rate of force change (increase in force) reached above 10 N/s after the target bars turned green (start cue) and the first time point at which the rate of force change (decrease in force) reached below 10 N/s after the target bars turned red (end cue), respectively. To include trials during which the task was performed as instructed and to remove trials with accidental contraction during the rest period, we excluded following trials from the subsequent analysis: (1) trials with force onset below 100 ms or above 1000 ms, (2) trials with force offset below 100 ms or above 1000 ms, and (3) force generation (> 10 N/s) during a period of 4000 ms from 1000 to 5000 ms with respect to the end cue. 46 trials (0.019%) were excluded from the following analysis.

We calculated mean force error (MFE) in percent MVF and coefficient of variation (CV) of force during the last 1000 ms of the contraction period, considering the transition phase from the force onset to the time of the exerted force being stable around the target force level^35^.

EEG data were analyzed using EEGLAB (http://sccn.ucsd.edu/eeglab/)^42^. They were first band-pass filtered between 1 and 45 Hz and then divided into epochs. The epoch window was defined as − 2000 to 6000 ms with respect to the force onset. We removed epochs contaminated with electrical activity exceeding ± 100 μV. Event-related spectral perturbation (ERSP) was computed between 3 and 45 Hz using Morlet wavelet transforms with 3 cycles at the lowest frequency and 9 cycles at the highest frequency. The values were normalized to a baseline, which was defined as − 1500 to − 500 ms with respect to force onset, and expressed in decibel (dB) units.

### Statistical analysis

The effects of group (yips vs. control) and visual gain (low vs. high) on ERD/ERS were evaluated using a bootstrap method within EEGLAB^43^. This analysis was performed for C3 and C4 electrodes separately. False discovery rate (FDR) correction was applied to correct for multiple comparisons^44^. Behavioral variables (MFE and CV) were analyzed with R (R Development Core Team). They were entered into a mixed-design analysis of variance (ANOVA) to examine the effects of group and visual gain. The data were log-transformed when not normally distributed. A significant level was set at α < 0.05.

## Results

### Event-related spectral perturbation

**Figure 2:**
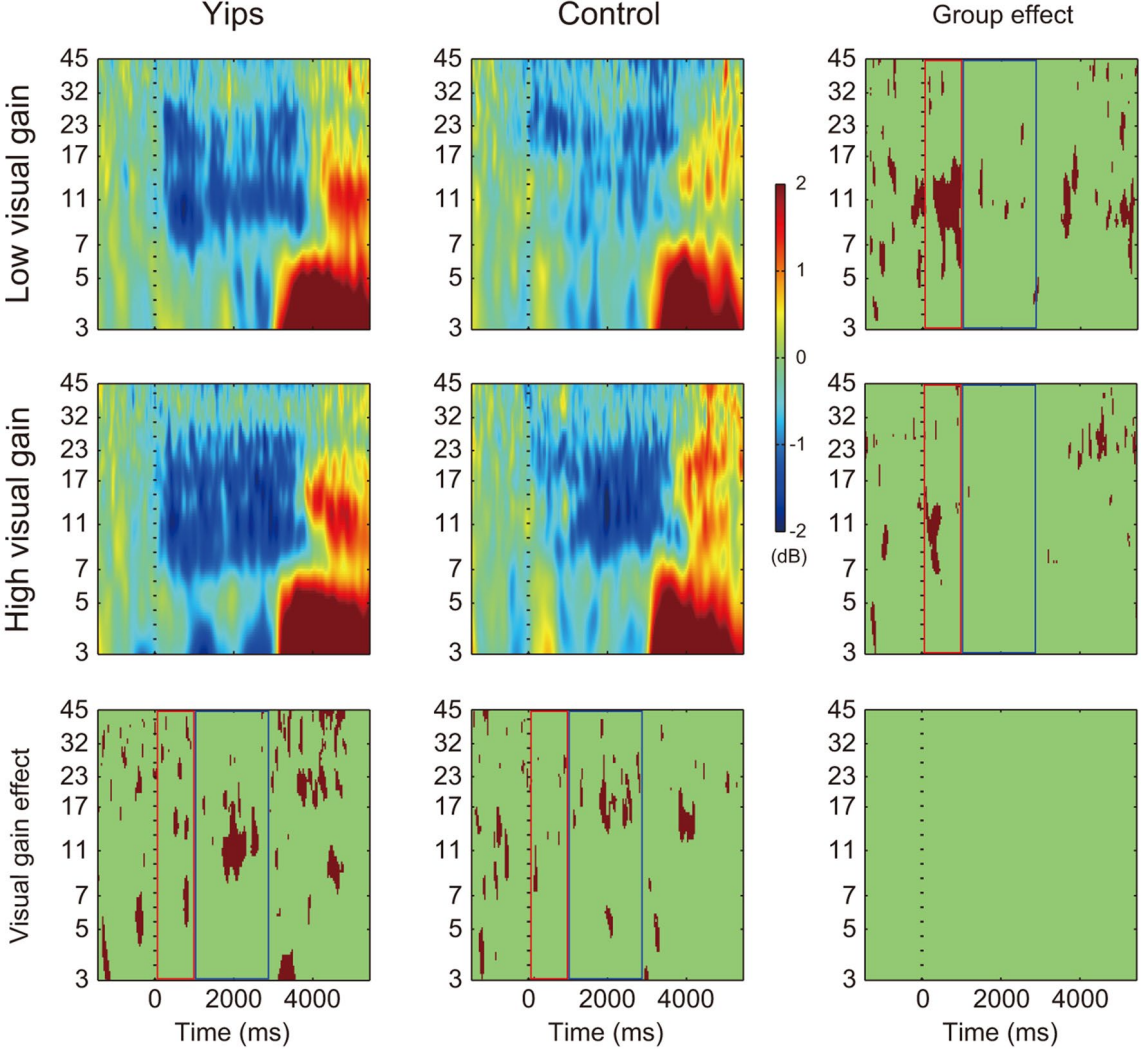
Grand average event-related spectral perturbation at C3 electrode. Dashed vertical line presents force onset. The color scale indicates the relative change from a baseline (− 1500 to − 500) in decibel (dB), with red being positive values and blue negative values. The last column indicates the area of statistically significant difference between groups, and the last row indicates the area of statistically significant difference between low and high visual gains (*p* < 0.05 with FDR correction, shown in brown). The plot at the bottom right presents the statistically significant interaction between group and visual gain. The red and blue squares indicate the force increase phase and force control phase, respectively.

**Figure 3:**
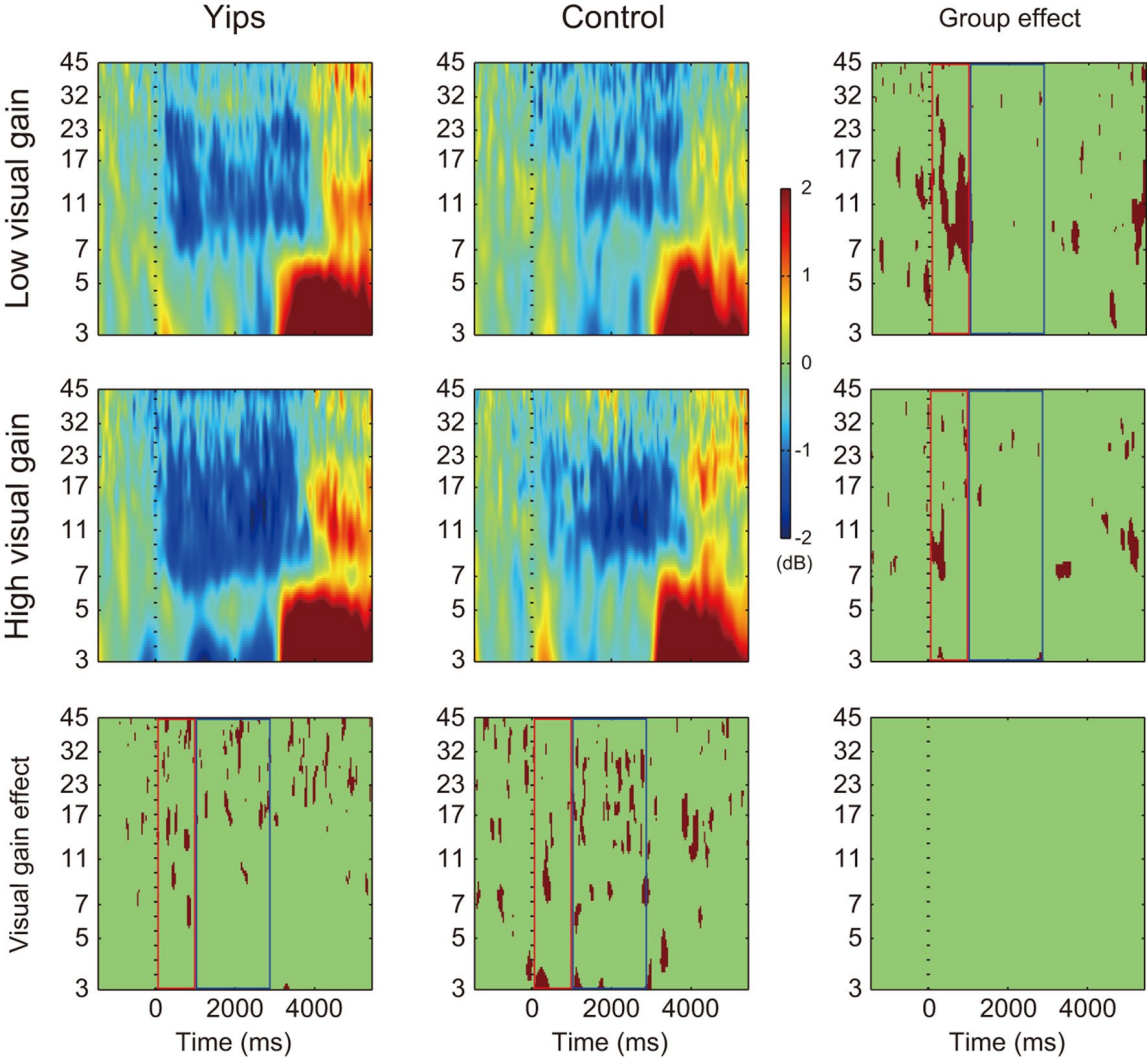
Grand average event-related spectral perturbation at C4 electrode. Dashed vertical line presents force onset. The color scale indicates the relative change from a baseline (− 1500 to − 500) in decibel (dB), with red being positive values and blue negative values. The last column indicates the area of statistically significant difference between groups, and the last row indicates the area of statistically significant difference between low and high visual gains (*p* < 0.05 with FDR correction, shown in brown). The plot at the bottom right presents the statistically significant interaction between group and visual gain. The red and blue squares indicate the force increase phase and force control phase, respectively.

Grand average ERSP time–frequency plots at C3 and C4 electrodes are presented in Figs. 2 and 3, respectively. ERD is presented with negative values (blue) while ERS is presented with positive values (red). At both electrodes, ERD can be observed in the alpha to beta range after force onset, which was followed by ERS in the same range after force offset. The last column shows the area of significant differences in ERD/ERS between athletes with yips and controls (group effect). The last row shows the area of significant differences in ERD/ERS between low and high visual gains (visual gain effect). Their interaction is presented at the bottom right. Comparison of ERSP at C3 electrode between groups revealed that the yips group showed a significantly greater alpha-band ERD in the early phase of task during which force was increased to match the target force level (force increase phase) at both low and high visual gain conditions. This difference was mostly absent in the later phase during which force was controlled around the target force level (force control phase). Also, they showed a significantly greater ERS in alpha and/or beta bands after force offset at both low and high visual gain conditions as compared to controls. When compared between two visual gains, beta-band ERD during the force control phase and alpha/beta-band ERS following the force offset were significantly greater at high than low visual gain in both groups. There was no significant interaction between group and visual gain. Similar significant differences were present for data at C4 electrode.

### Behavioral parameters

**Figure 4:**
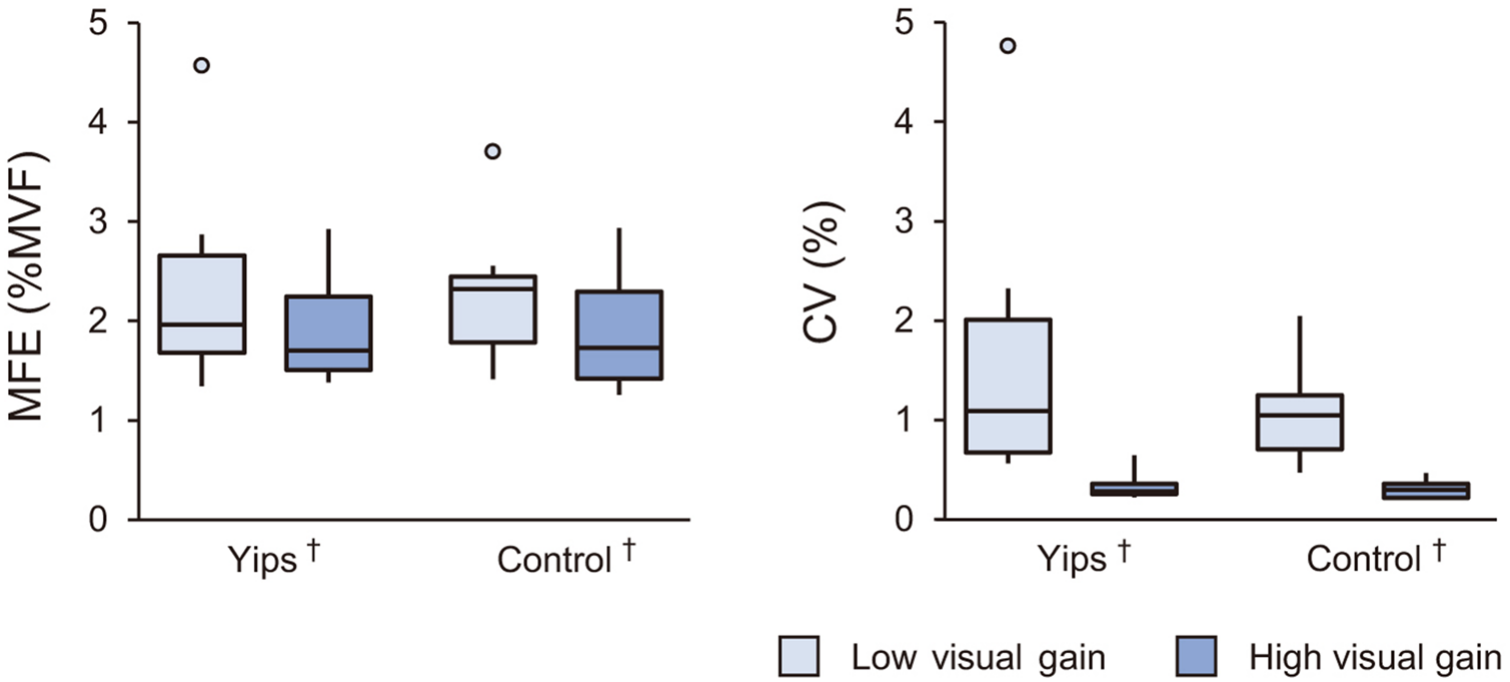
Box plots of mean force error (MFE) and coefficient of variation (CV) of force. The median and interquartile ranges are presented with whiskers representing the maximum and minimum values. Small circles show outliners. The daggers indicate a significant main effect of visual gain.

Figure 4 shows results of MFE and CV of force. A mixed-design ANOVA revealed a main effect of task for the MFE (F(1,18) = 56.2, *p* < 0.001, η^2^ = 0.52) and also for the CV of force (F(1,18) = 9.7, *p* = 0.01, η^2^ = 0.065). There was no significant main effect of group or interaction between group and visual gain.

## Discussion

This study compared event-related sensorimotor cortical oscillatory activity during a precision force control task between athletes with yips and age-, sex-, and years of experience-matched controls. We found that alpha-band ERD over the ipsilateral and contralateral sensorimotor areas was greater during the force increase phase (movement initiation) in yips than control group, regardless of the precision level required in the task. There was no apparent difference in ERD during the force control phase (controlling force around the target force level). Furthermore, alpha/beta-band ERS following force offset was greater in yips than control group. These findings suggest alteration of sensorimotor cortical oscillatory activity in a task requiring fine motor control in athletes with yips.

Alpha and beta-band ERD has been consistently reported to reflect increased activation of the sensorimotor cortical areas during preparation or execution of movements^29,33,35,45^. Furthermore, an increase in ERD was found to reflect increased corticospinal excitability and reduced intracortical inhibition in a TMS study^46^. Thus, our finding of enhanced alpha-band ERD could indicate that sensorimotor cortical activity as well as corticospinal excitability were increased while intracortical inhibition was reduced particularly when increasing force to match the target level force in athletes with yips. This is in line with previous brain imaging and TMS studies reporting increased motor cortical excitability and decreased intracortical inhibitory system in individuals with task-specific focal dystonia^19–26^. Particularly, Sohn and Hallett found an increase in amplitude of motor-evoked potential recorded from the little finger muscle just after index finger movement onset (3–80 ms after electromyographic onset) in individuals with focal hand dystonia^25^. Also, intracortical inhibition was found to be impaired during movement initiation. Specifically, in a rhythmic index finger flexion task, control subjects showed a decrease in intracortical inhibition acting on the index finger and an increase in intracortical inhibition on the thumb muscle during the electromyographic burst; however, these modulations were not observed in individuals with focal hand dystonia^24^. Moreover, in the following study that used a force control task with the index finger, individuals with focal hand dystonia showed a reduced intracortical inhibition on the thumb muscle during movement initiation but not during tonic contraction^22^. Hence, the increased ERD in athletes with yips may be attributed to the reduced intracortical inhibition acting on task-irrelevant muscles. However, it is currently unclear whether the yips is a pure form of task-specific focal dystonia, although some research argues that it is^13,14^. Further studies are needed to better understand the neurophysiological mechanisms behind the yips and whether or not it is on a spectrum of task-specific focal dystonia. In any case, the cortical oscillatory response is enhanced during movement initiation in athletes with yips.

The important question to ask here is “why the difference in ERD was observed mainly in alpha band,” even though both alpha- and beta-band rhythms were desynchronized more or less similarly. One potential explanation is a relatively stronger association of the alpha-band oscillatory activity with motor cortical excitation and inhibition. Specifically, Sauseng and colleagues examined cortical oscillatory activity immediately before applying a TMS pulse over the motor cortex and found that motor evoked potential was elicited more easily when alpha-band power over the sensorimotor cortex was lower^47^. No such effect was observed for the other frequency bands (delta, theta, beta, or gamma). Also, alpha-band activity over the bilateral sensorimotor cortices was found to increase when a planned movement was required to be suppressed^48^. Again, this effect was highly frequency-band specific. It is, therefore, possible that the *motor* cortical alteration of athletes with yips was mainly reflected by alpha-band activity. Another possible explanation for this band specificity is voluntary attention to the body part that is used during task performance, as a growing body of evidence shows that alpha-band activity decreases in task-related cortical areas to facilitate sensory processing and increase in task-unrelated areas to suppress distracting inputs. For instance, it is well-known and established that voluntary orientation of visuo-spatial attention is accompanied by a decrease in occipital alpha-band activity^49,50^. In a similar manner, voluntarily orienting attention to a body part has been reported to decrease alpha-band activity over the somatosensory areas^51,52^. It can, hence, be hypothesized that greater alpha-band ERD observed in the present study was ascribed to voluntary orientation of attention to the index finger and thumb during the precision force control task, in order to facilitate top-down control. This hypothesis further seems to be in line with reinvestment theory recently proposed to explain the etiology of the yips^16,17,53^. In the course of motor skill learning, performance will become more and more stable and turn into automatic control; however, when modifications of acquired motor skill are required because of various intrinsic and extrinsic factors (e.g., injury and change in technic or equipment), s/he needs to provoke reinvestment, an attempt to change the stable and automatic motor skill by consciously controlling own movement with the use of explicit knowledge (i.e., breaking down the automatic motor skill into smaller chunks as in the early learning stage)^10,53,54^: this attempt could result in a state where the acquired motor skill is vulnerable to disruption, and repeated attempts of unsuccessful performance can consolidate a deteriorated movement control^10,53^. This theory is supported by results of a previous questionnaire study showing a tendency in athletes with yips to consciously control their movements^1^. Yet, it is also possible that the athletes begin to consciously control their movements to prevent an error after acquisition of the yips. Nevertheless, the increase in alpha-band ERD could have been driven by a greater degree of conscious movement control by allocating the attention to the body part used for the task. How reinvestment is involved in the yips appears to need further investigations.

In addition to the increased ERD, we found that post-movement ERS was greater in athletes with yips as compared to controls. The post-movement ERS has been considered to reflect an active inhibition of the motor cortex and its related networks following the termination of movement^55–57^, and a previous study has reported that the ERS tended to be greater after complex than simple movements^58^. Thus, although there was no difference in task performance between yips and control groups, the fine force control task could have been difficult for athletes with yips, and they might have devoted the greater effort to the task, consequently resulting in the greater post-movement ERS. However, we did not find a difference in the ERD during the force control phase between two groups, which contradicts previous studies’ finding of greater ERD in a more complex task^35,58,59^. Moreover, because the purpose of this study was to examine the cortical oscillatory activity during a fine motor task, we did not adopt a motor task that induces the yips, in order to control the movement during the task (e.g., force and postural position), similar to previous studies on task-specific focal dystonia^22,26,30,32^. This could underlie the insignificant difference in the task performance and have possibly obscured the potential difference in the cortical activity during the force control phase. The occurrence of the yips is highly movement specific and is dependent on the situation (e.g., competition), which makes it difficult to record neurophysiological data during the affected movements while controlling for confounding factors (e.g., movement). This issue needs to be resolved in future studies with technological progress to advance knowledge of the yips.

In regards to the effect of visual gain, we found that beta-band ERD/ERS was greater during high than low visual gain condition for both yips and control groups. At the higher visual gain, the subject can see force deviations from a target more clearly, and this consequently forces the subject to control the force more precisely. Similar to previous studies^35,59^, the increase in task demands most likely enhanced the motor cortical oscillatory response.

The yips has received considerable attention recently as famous professional athletes not only in golf but also in other sports, such as baseball, basketball, and tennis, have confessed that they have suffered from the yips at some point during their career. However, because clinical data are limited to few case studies^60,61^, evidence-based treatment for the yips has not been established yet. Although further studies are necessary, our findings indicate that athletes with yips may benefit from avoiding allocating too much attention to the affected movements and also from learning such strategies. Real-time visual feedback of cortical activity may help the learning process. In addition, non-invasive brain stimulation to modulate the intracortical inhibitory system^62^ may be applicable to the yips. Nonetheless, randomized clinical trials are essential for the valid assessment of treatments and thus needed to improve the evidence regarding the treatment of the yips.

There are several limitations that should be acknowledged. First, we used only two electrodes to record EEG over the sensorimotor areas. Recording and analysis of high-density EEG could have strengthened our discussion, especially on the conscious top-down control of movements (e.g., frontal lobe). Second, only an affected hand was examined in this study, and data during the task using a hand contralateral to the affected side may have provided additional information. Future studies need to clarify brain activity associated with the contralateral hand movements. Third, although the occurrence of the yips is highly movement specific and is dependent on the situation, recording of electromyogram may have clarified abnormal muscle activity during the task. Finally, subjects of this study were baseball and badminton players; therefore, the findings may not be generalized to golfers or athletes in other sports (e.g., runners).

To sum up, our findings indicate alteration of sensorimotor cortical oscillations during precision force control in athletes with yips. Especially, alpha-band ERD was greater in these individuals than controls when increasing force output to match a target but not when adjusting the force at around the target. The enhanced ERD is suggested to be attributed to dysfunction of inhibitory system or increased allocation of attention to the body part used during the task. Our findings provide new evidence that sensorimotor cortical oscillatory response is increased particularly during movement initiation in athletes with yips.

## Data Availability

The datasets generated during and/or analyzed during the current study are available from the corresponding author on reasonable request.
